# Nodular nuances: A case of progressive cutaneous thickening

**DOI:** 10.1016/j.jdcr.2025.09.005

**Published:** 2025-09-19

**Authors:** Lauren James, Ryan Geist, Danielle Johnston, Greta Nebeker, Jonathan Crane

**Affiliations:** a Dermatology Department, Campbell University/Sampson Regional Medical Center Dermatology Residency Program, Wilmington, North Carolina; b Dermatology Department, Campbell College of Osteopathic Medicine, Buies Creek, North Carolina

**Keywords:** bacterial infection, fibrohistiocytic proliferation, Fite stain, foamy histiocytes, globi, grenz zone, Hansen’s disease, histopathology, immunohistochemistry, lepromatous leprosy, multibacillary leprosy, *Mycobacterium leprae*, perineural inflammation, Virchow cells

## Case description

**Fig 1:**
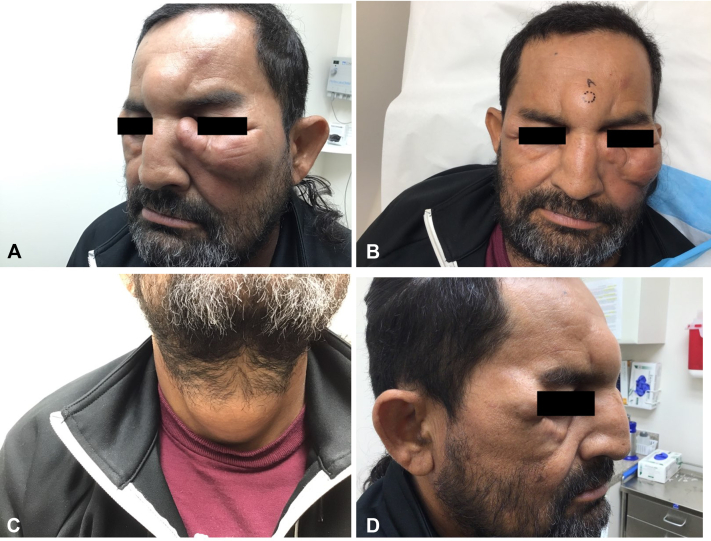
**A-C,** Skin-colored nodules involving the glabella and periocular region. **D,** Skin-colored nodule involving the anterior neck.

A 50-year-old male with no relevant medical history presented with asymptomatic head, neck, and extremity lesions which had been slowly growing over the last 2 years. The patient moved to the United States 20 years prior from Honduras and does not follow regularly with primary care. Physical examination revealed numerous skin-colored nodules on the glabella (Fig 1, *A*), bilateral periocular region (Fig 1, *A*-*C*), bilateral cheeks (Fig 1, *A*-*C*), anterior neck (Fig 1, *D*), and right elbow. The areas of diffuse thickened skin revealed a “doughy” texture on palpation. Neurologic examination revealed mild neuropathy involving the bilateral distal extremities. Punch biopsies from the forehead and left malar cheek revealed a dense, sheet-like infiltrate of large histiocytes with foamy, vacuolated, gray-blue cytoplasm, some of which contained well-circumscribed amphophilic globular inclusions. A cell-poor zone within the papillary dermis, consistent with a grenz zone, separated the infiltrate from the overlying thinned epidermis. In the mid to deep reticular dermis, the infiltrate exhibited frequent periadnexal, perineural, and perivascular extension. No well-formed granulomas were identified. These lesional cells demonstrated immunoreactivity for CD68 and CD163 and were negative for S100 and CD1a.

**Question 1: What is the most likely diagnosis?**

A. Scleromyxedema
B. Cutaneous T cell lymphoma
C. Sarcoidosis
D. Lepromatous leprosy
E. Cutaneous leishmaniasis

## Case discussion

D. Lepromatous leprosy – Correct. Lepromatous leprosy is the most severe form of Hansen’s disease, an infectious condition caused by *Mycobacterium leprae,* an acid-fast bacillus with a predilection for skin and peripheral nerves. It represents the multibacillary end of the leprosy spectrum, characterized by defective cell-mediated immunity (CMI) against *M. leprae*, leading to widespread dissemination of bacilli. Lepromatous leprosy features diffuse, symmetrical skin lesions with many bacteria due to weak cell-mediated immunity and a dominant Th2 immune response. In contrast, tuberculoid leprosy demonstrates few well-defined lesions and significant neuropathy, driven by a strong Th1 immune response.^1^

Clinically, patients present with numerous, symmetric, poorly demarcated macules, papules, nodules, or diffuse skin infiltration, often with “leonine facies” due to facial skin thickening. Sensory loss typically occurs late in the disease course and is less pronounced initially, but progressive nerve involvement leads to glove-and-stocking anesthesia and deformities.^2^ Mucosal involvement (notably nasal and oral) and madarosis are also common findings.

Histologically, lepromatous leprosy presents with a diffuse infiltrate of foamy (Virchow) macrophages packed with numerous acid-fast bacilli, minimal lymphocytic response, and absence of well-formed granulomas.^3^ The dermal infiltrate extends around neurovascular bundles and adnexal structures. Relevant stains for lepromatous leprosy include Fite-Faraco and Ziehl-Neelsen, which are used to identify numerous acid-fast bacilli inside macrophages. Immunophenotyping shows mainly CD68+ macrophages with few CD4+ T cells, indicating a Th2-skewed immune response. There is also a significant reduction of CD1a+ cells in lesions compared to tuberculoid leprosy.^4^

The recommended treatment regimen for lepromatous leprosy, as endorsed by the World Health Organization (WHO) is a multidrug therapy (MDT) consisting of rifampicin (600 mg once monthly supervised), clofazimine (300 mg once monthly supervised and 50 mg daily self-administered), and dapsone (100 mg daily self-administered) for at least 12 months.^5^ Prognosis is favorable with early diagnosis and adherence to MDT, but delayed treatment can result in irreversible nerve damage, deformities, and disability.

## Conflicts of interest

None disclosed.
